# Prevalence and pattern of migration intention of doctors undergoing training programmes in public tertiary hospitals in Ekiti State, Nigeria

**DOI:** 10.1186/s12960-022-00772-7

**Published:** 2022-10-27

**Authors:** Adebowale Femi Akinwumi, Oluremi Olayinka Solomon, Paul Oladapo Ajayi, Taiwo Samuel Ogunleye, Oladipupo Adekunle Ilesanmi, Adedayo Olufemi Ajayi

**Affiliations:** 1 Department of Community Medicine, Faculty of Clinical Sciences, College of Medicine, Ekiti State University, Ado Ekiti, Nigeria; 2 Department of Community Medicine, Ekiti State University Teaching Hospital, Ado Ekiti, Nigeria; 3 Department of Community Medicine, Federal Teaching Hospital, Ido-Ekiti, Nigeria; 4 Department of Agricultural Extension and Rural Development, Obafemi Awolowo University, Ife, Nigeria

**Keywords:** Migration intention, Doctors, Residency and internship training, Tertiary hospitals, Nigeria

## Abstract

**Background:**

Emigration of Nigerian doctors, including those undergoing training, to the developed countries in Europe and Americas has reached an alarming rate.

**Objective:**

This study aimed at assessing the prevalence, pattern, and determinants of migration intention among doctors undergoing residency and internship training programmes in the public tertiary hospitals in Ekiti state, Nigeria.

**Methods:**

This was a cross-sectional study using a quantitative data collected from 182 doctors undergoing residency and internship training at the two tertiary hospitals. An adapted semi-structured questionnaire was used to collect information on migration intention among the eligible respondents. Univariate, bivariate and multivariate data analyses were done. The level of significance was determined at *p*-value < 0.05.

**Results:**

Majority (53.9%) of doctors undergoing training were between 30–39 years, and the mean age was 33.2 ± 5.7 years, male respondents were 68.1%, and 53.8% of the respondents were married. The proportion of doctors undergoing training who had the intention to migrate abroad to practice was 74.2%. A higher proportion of the internship trainees, 79.5%, intended to migrate abroad to practice while the proportion among the resident doctors, was 70.6%. Among the respondents who intended to migrate abroad to practice, 85(63%) intend to migrate abroad within the next 2 years, while the preferred countries of destination were the United Kingdom 65(48.2%), Canada 29 (21.5%), Australia 20 (14.8%) and the United States 18(13.3%). Seventy percent of respondents who intend to migrate abroad had started working on implementation of their intention to migrate abroad. The majority of the junior resident doctors, 56(72.7%), intend to migrate abroad compared with the senior resident doctors, 21(27.3%), (*χ*^2^ = 14.039; *p* < 0.001). The determinants of migration intention are the stage of residency training and level of job satisfaction.

**Conclusion:**

There is a high prevalence of migration intention among the doctors undergoing training in the public tertiary hospitals in Ekiti State, Nigeria, with the majority already working on their plans to migrate abroad. Doctors undergoing training who are satisfied with their job and those who are in the senior stage of residency training programme are less inclined to migrate abroad.

**Recommendations:**

The hospital management in the tertiary hospitals should develop retention strategies for human resources for health, especially doctors undergoing training in their establishment, to avert the possible problems of dearth of specialists in the tertiary health facilities. Also, necessary support should be provided for the residency training programme in the tertiary health institutions to make transition from junior to senior residency stage less strenuous.

## Introduction

### Background of the study

Emigration of Nigerian doctors to the developed countries in Europe and Americas has reached an alarming rate [1, 2]. The phenomenon popularly referred to as brain drain [3, 4] has posed serious challenges to the human resources for health in Nigeria. Resident doctors undergoing specialist training in the country’s many tertiary hospitals have constituted significant proportion of physicians who had left or have intention to leave Nigeria for other nations of the world. This is also obtainable among the graduate medical doctors (house officers) doing their mandatory 1-year internship in the tertiary hospitals across the nation. These young doctors form the critical mass that should be developed through training and mentorship to replace older ones in the medical practice.

Physician migration is an age-long phenomenon [3, 5]. It has been recognized as a major concern for both the donor countries, usually low- and middle-income countries and the recipient countries, the developed countries of North America, Europe and Australia due to a higher number of doctors migrating or intending to migrate compared to the past [3, 6]. The World Health Organization (WHO) formulated a Global Code of Practice on the International Recruitment of Health Personnel as a response to the emigration of doctors and other health professionals from low- and middle-income countries (LMICs) to high-income countries [7]. The recent surge in migration is due to the widening gap between demand and supply of physicians in developed countries [8].

It is stated that turnover intention can be used as a valid proxy for measuring definite labour turnover [9, 10], and by extension, migration intention for actual migration. Researchers have described turnover or migration intention as “the final step in the decision-making process before a person actually leaves a workplace or its country of primary residence, in which members actively consider quitting and searching for alternative jobs or professions or determine to move to another country” [11, 12].

Retention of healthcare workers is a significant concern due to cost of hiring and training of new workers. Besides, shortage of health workforce is detrimental to healthcare system performance and services. According to the WHO, healthcare labour shortages are common globally; however, this phenomenon is crucial in countries where healthcare performance indicators are the worst [7].

In 2005, Mullan et al. reported that international medical graduates constitute between 23 and 28% of physicians in the high-income countries such as the United States, United Kingdom, Canada, and Australia, and the lower-income countries such as India, the Philippines, Pakistan, South Africa and Nigeria supply between 40 and 75% of these international medical graduates [3]. In fact, out of the 20 countries with the highest emigration factors, nine are in the sub-Saharan Africa or the Caribbean [3].

The Nigerian health sector has in the recent times faced with massive migration of doctors to the foreign lands. About 40% of all physicians from sub-Saharan African region employed in the United States were trained in Nigeria [5]. In recent time, there were clear evidence of the unprecedented level of physician migration intention and actual migration by many screaming headlines on the medical brain drain conundrum in the prominent Nigerian national dailies and international media [1, 13, 14]. This situation has impacted negatively on the availability and quality of health care services and delivery to the people. Migration of physicians has been at considerable economic cost to the nation, it has depleted human resources for health, reduced the effectiveness of health care delivery and the morale of the remaining workforce who are now overworked [1].

The working environment of doctors in Nigeria is not optimal. Necessary medical equipment are in short supply in many public health institutions at the primary, secondary and the tertiary levels. Majority of doctors, especially doctors under training working in public health institution in Nigeria are dissatisfied with their job [2, 15, 16]. There is also challenge of inter-professional rivalry and poor harmonious working relationships among the various professional groups in the health sector especially, the secondary and tertiary health institutions [17–19]. These situations have led to many industrial actions in the past [2, 17–19]. These reasons and poor salaries, had prompted many doctors, especially young medical graduate and resident doctors to leave and many more currently having intention to migrate to other countries to work.

In order to strengthen and regulate residency training programme in the country, the Federal Government of Nigeria enacted a law, Medical Residency Training (MRT) Act 2017 [20] with the implementation started in all federal tertiary hospitals in 2020. Many tertiary hospitals owned by state governments are yet to domesticate and implement the MRT Act. The effect of this new policy and legislation on migration intention and brain drain among doctors undergoing training in these tertiary hospitals are yet to be ascertained.

Research evidence has shown that turnover or migration intention was the strongest predictor of actual turnover or migration among healthcare personnel [10]. It is believed that early detection of employee’s migration intention would be greatly helpful to resolve the problem of exodus before it exacerbates. While there are some studies on the migration of physicians from sub-Saharan Africa to other developed countries [3, 5, 9, 21, 22], there are fewer studies on the subject of migration among doctors undergoing trainings in the African region [23]. However, studies on migration intention among doctors undergoing training in Nigeria are almost non-existent.

With the hope of ascertaining the level of migration intention among doctors undergoing residency and internship training in the public tertiary hospitals, this study sought to provide empirical evidence for health care managers in the two institutions and the state government which could be used to develop strategies to address major drivers of job dissatisfaction among these doctors. Also, information to be provided on the pattern of migration intention among this category of young doctors will assist policy-makers and the government agencies concerned to nip in the bud exodus of doctors with timely interventions. This study thus aimed at assessing the prevalence, pattern and determinants of migration intention among doctors undergoing residency and internship training programmes in the public tertiary hospitals in Ekiti State, Nigeria.

## Research methodology

### Area of study

This study was conducted in the two public tertiary hospitals in Ekiti State—Ekiti State University Teaching Hospital (EKSUTH), Ado-Ekiti and the Federal Teaching Hospital, Ido Ekiti (FETHI). The EKSUTH is located in Ado-Ekiti, the capital of Ekiti State, in Ekiti Central Senatorial district, while the FETHI is located in Ido Ekiti, Ekiti North Senatorial district. Ekiti State was created in 1996. Ekiti State has a land mass of 5887.890 sqm and a projected population of 3 885 827 in 2021. The state has 3 tertiary hospitals (2 public and 1 private), several general hospitals, comprehensive and primary health centres. The two public tertiary hospitals were selected for the study because they are accredited for postgraduate residency training programme. Also, they have doctors undergoing residency and internship training programmes drawn up from many parts of the country.

The two public tertiary hospitals are more than 150-bed capacity consisting of adult and children medical and surgical wards, children and adult emergency wards, psychiatry wards, maternity complex wards (comprising gynaecology ward, antenatal ward, post-natal ward and special care baby unit). They have clinics that are being run at different time of the days and hours. Each has more than 1400 staff, made of doctors, nurses, pharmacists, laboratory scientists and technicians and other administrative and supporting staff.

Both the EKSUTH Ado-Ekiti and FETHI Ido-Ekiti have many medical and surgical departments where resident doctors undergo residency training programme. There are 82 resident doctors and 24 house officers in EKSUTH in April 2020, while the FETHI has a total of 174 resident doctors and 50 house officers in same month.

### Study design

This was a cross-sectional study using quantitative method of data collection.

### Study population

The study populations were the doctors undergoing residency and internship training programmes at public teaching hospitals in Ekiti State. Those included were all resident doctors undergoing residency training programme, and the graduate medical doctors (house officers) who have spent minimum of 1 month into their 1-year mandatory internship training in public tertiary hospitals in Ekiti State. Excluded from the study were doctors undergoing residency and internship training programmes who were on leave of absence during the study period, on outside posting in another tertiary hospital outside Ekiti State or were critically ill.

### Sample size determination

The minimum sample size was determined using the formula for calculating sample size for the single proportion in a known finite population. The total population of doctors under training in both public tertiary health institutions in Ekiti State where the study was conducted is 330. The sample size was calculated with *Zα*, standard normal deviate corresponding to 95% confidence level, proportion of Portuguese junior doctors who were considering working abroad within the next 10 years (55.0%) [24], adjusted for a sample size in a finite population less than 10 000, and corrected for anticipated non-response rate of 10%, the minimum sample size for the study was rounded up to 200.

### Sampling procedure

A systematic sampling technique was used to select respondents from the list of resident doctors and house officers in the two public tertiary health institutions in Ekiti State. The nominal roll of all doctors under the residency and internship training was used as the sample frame. A proportionate sample of resident doctors and house officers from all the training departments on the nominal rolls in the two public tertiary health institutions was sampled. On the average, a sampling interval of 2 was used during the systematic sampling procedure and where this was not feasible, simple random sampling by balloting was used to select eligible respondents.

### Outcome and explanatory variables

The primary outcome measures for this study include the migration intention of doctors under training to practice abroad, categorized into “yes” and “no”; and overall level of job satisfaction of the doctors under training in the two tertiary hospitals categorized as “satisfied”, “undecided” and “unsatisfied”. Explanatory variables include socio-demographic characteristics, training characteristics, number of years in practice, and other factors influencing migration intention among the doctors under training.

### Research instrument

The study instrument that was used in this study is a semi-structured questionnaire, adapted from validated questionnaire used in a previous study on post-graduation migration intentions among medical graduates in Pakistan [25]. This was used to collect information about socio-demographic characteristics, views of resident doctors and house officers about their intention to migrate, purpose for their migration intention, and their influencing factors, level of job satisfaction, among others.

### Data analysis techniques

Data were entered using Microsoft Excel 2010 version into pass-worded personal computer and analysed using STATA statistical software version 15. Univariate analysis was used to generate descriptive statistics and bivariate analysis-Chi square was deployed to determine relationship among intention to migrate (categories) and other categorical variables. Binary logistic regression was done to identify determinants of intention to migrate abroad among the respondents. The level of significance was determined at *p*-value < 0.05.

### Data collection procedure

Two graduate research assistants were involved in the data collection. One-day training was organized for the research assistants on the content and administration of the research instruments. It included practical demonstrations in the class room and on the field. The data were collected in October 2020.

## Results

Two hundred questionnaires were administered to eligible respondents, 182 were retrieved and completely filled, given a response rate of 91%.

The socio-demographic and training characteristics of doctors undergoing training in public tertiary hospitals in Ekiti State are shown in Table 1. The proportion of doctors undergoing training at the public tertiary hospitals within the age group 29 years and below were 56 (30.7%) and 30–34 years 54 (29.7%). The mean age of the respondents was 33.2 ± 5.7. Male doctors undergoing training were 124 (68.1%) and 98 (58.3%) of the respondents were married. Majority 166 (91.2%) of the respondents were Yoruba and 170 (93.4%) were Christians. Sixty percent of the respondents were into residency training programme.

**Table 1:** Socio-demographic and training characteristics of doctors undergoing training in Public Tertiary Hospitals in Ekiti State

Socio-demographic characteristics of health care workers	Frequency (*n* = 182)	Percentage %
Age (years)		
≤ 29	56.0	30.7
30–34	54.0	29.7
35–39	44.0	24.2
40 and above	28.0	15.4
Mean age:	33.2 ± 5.7	
Range (age)	22–49	
Sex		
Male	124.0	68.1
Female	58.0	31.9
Marital status		
Single	83.0	45.6
Married	98.0	53.8
Divorced	1.0	0.6
Ethnicity		
Yoruba	166.0	91.2
Igbo	10.0	5.5
Hausa	1.0	0.6
Others	5.0	2.7
Religion		
Christianity	170.0	93.4
Islam	11.0	6.0
Other	1.0	0.6
Self-rated level of economic status		
Lower	5.0	2.7
Lower middle	81.0	44.5
Upper middle	90.0	49.5
Upper	6.0	3.3
Type of training undergoing		
Internship	73.0	40.1
Residency	109.0	59.9
Tertiary hospital undergoing training		
FETHI	118.0	64.8
EKSUTH	64.0	35.2
Department undergoing training		
Medically related	109	59.9
Surgically related	73	40.1

The prevalence of migration intention among doctors undergoing residency and internship training programmes in public tertiary hospitals in Ekiti State is shown on Table 2. The proportion of doctors undergoing training that had intention to migrate abroad to practice was 135 (74.2%). A higher proportion of the internship trainees 58 (79.5%) had intention to migrate abroad to practice than the resident doctors 77 (70.6%). Among the resident doctors, higher proportion of junior resident doctors 56 (83.6%) had intention to migrate abroad to practice than their senior counterparts 21 (50%).

**Table 2:** Prevalence of migration intention among doctors undergoing residency and internship training programmes in public tertiary hospitals in Ekiti State

Prevalence of migration intention among doctors undergoing training	Frequency	Percentage %
Intention to migrate abroad as a doctor to practice	(*N* = 182)	
Yes	135.0	74.2
No	47.0	25.8
Internship trainees	(*n* = 73)	
Yes	58.0	79.5
No	15.0	20.5
Resident doctors	(*n* = 109)	
Yes	77.0	70.6
No	32.0	29.4
Junior resident doctors	(*n* = 67)	
Yes	56.0	83.6
No	11.0	16.4
Senior resident doctors	(*n* = 42)	
Yes	21.0	50.0
No	21.0	50.0
EKSU	(*n* = 64)	
Yes	49.0	76.6
No	15.0	23.4
FETHI	(*n* = 118)	
Yes	86	72.9
No	32	27.1

The pattern of migration intention among doctors undergoing residency and internship training in public tertiary hospitals in Ekiti state is shown in Table 3. Among the doctors undergoing training who had intention to migrate abroad to practice, 63% intend to migrate abroad within the next 2 years, while about 29 (21.5%) of them intend to migrate abroad between 3–5 years from year of study. The proportion of doctors undergoing training who intended to migrate abroad to pursue further specialty training at their country of destination was 104 (77.0%). The countries of destination preferred by the majority of the respondents who intend to migrate abroad were United Kingdom 65 (48.2%), Canada 29 (21.5%), Australia 20 (14.8%) and United States 18 (13.3%). Seventy percent of doctors in training who intend to migrate abroad had started working on their intention to migrate abroad.

**Table 3:** Pattern of migration intention among doctors undergoing residency and internship training in public tertiary hospitals in Ekiti State

Pattern of migration intention among doctors undergoing training	Frequency (*n* = 135)	Percentage %
When doctors in training intend to migrate abroad		
Within next 1 year	29.0	21.5
1–2 years	56.0	41.5
3–5 years	29.0	21.5
6–10 years	6.0	4.4
Greater than 10 years	1.0	0.7
Don’t know	14	10.4
Purpose of doctors’ intention to migrate abroad		
For further specialty training	105.0	77.8
To work/practice as doctor without further specialty training	24.0	17.8
Don’t know	6.0	4.4
The preferred destination for doctors’ intention to migrate		
UK	65.0	48.2
Canada	29.0	21.5
Australia	20.0	14.8
USA	18.0	13.3
Gulf countries	2.0	1.5
Other	1.0	0.7
Doctors in training have started working on the intention to migrate abroad		
Yes	94.0	69.6
No	41.0	30.4
How long they have been working on their intention to migrate abroad	(*n* = 94)	
Before I graduated from medical school	14.0	14.9
Since I graduated from medical school	36.0	38.3
During my NYSC	5.0	5.3
After NYSC/working as medical officer	14.0	14.9
During my residency training	26.0	26.6
Plan to come back to Nigeria to practice after migrating abroad		
Yes	73.0	54.1
No	62.0	45.9

**Fig. 1:**
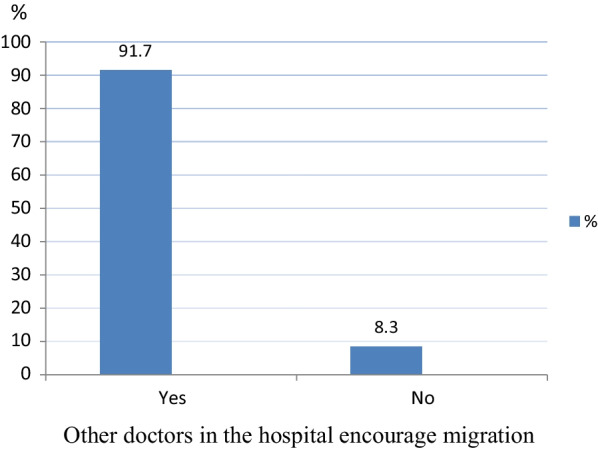
Influence of other doctors in the hospital in encouraging doctors undergoing training programmes to migrate abroad

The influence of other doctors in the hospital in encouraging doctors undergoing residency and internship training in public tertiary hospitals in Ekiti State to migrate abroad is shown in Fig. 1. Majority (91.7%) of doctors undergoing training programmes who intend to migrate abroad reported that other doctors in their hospital encourage them to migrate and train abroad.

The relationship between socio-demographic and training characteristics, and migration intentions of doctors undergoing residency and internship training in public tertiary hospitals in Ekiti State is shown in Table 4. Higher proportion of doctors undergoing training who were age 34 years and below, 89 (65.9%) intend to migrate abroad compared with those who were older, 46 (34.1%). The relationship is statistically significant; (*χ*^2^ = 6.581, *p* = 0.010). Similarly, the majority of the junior resident doctors, 56 (72.7%) intend to migrate abroad compared with the senior resident doctors, 21 (27.3%). The association is statistically significant; (*χ*^2^ = 14.039, *p* < 0.001). Other socio-demographic characteristics such as gender, religion, ethnicity, marital status, socio-economic status and training characteristics such as training institution and type do not have statically significant relationship with the migration intention of doctors undergoing training at the public tertiary hospitals in Ekiti State. Majority of the doctors undergoing training who were satisfied with their job do not intend to migrate abroad, 35 (74.5%) compared with those who were unsatisfied, 4 (8.5%) and undecided 8 (17.0%). This relationship is statistically significant (*χ*^2^ = 11.522; *p* = 0.003).

**Table 4:** Relationship between socio-demographic and training characteristics and migration intentions of doctors undergoing residency and internship training in public tertiary hospitals in Ekiti State

Socio-demographic factors	Intention to migrate abroad as a doctor to practice? *n* = 182 (%)			Statistics *χ*^2^
	Yes	No	Total	
Age group (years)				
≤ 34 years	89 (65.9)	21 (44.7)	110 (60.4)	*χ*^**2**^ = 6.581 *p* = 0.010* *df* = 1
35 years and above	46 (34.1)	26 (55.3)	72 (39.6)	
Sex				
Male	96 (71.1)	28 (59.6)	124 (68.1)	*χ*^**2**^ = 2.137 *p* = 0.144 *df* = 1
Female	39 (28.9)	19 (40.4)	58 (31.9)	
Ethnicity				
Yoruba	126 (93.3)	40 (85.1)	166 (91.2)	LR = 4.738 *p* = 0.094 *df* = 2
Igbo	7 (5.2)	3 (6.4)	10 (5.5)	
Others	2 (1.5)	4 (8.5)	6 (3.3)	
Religion				
Christianity	128 (94.8)	42 (89.4)	170 (93.4)	LR = 2.675 *p* = 0.263 *df* = 2
Islam	6 (4.4)	5 (10.6)	11 (6.0)	
Other	1 (0.8)	0 (0.0)	1 (0.6)	
Marital status				
Single	67 (49.6)	16 (34.1)	83 (45.6)	LR = 5.8157 *p* = 0.055 *df* = 2
Married	68 (50.4)	30 (63.8)	98 (53.9)	
Divorced	0 (0.0)	1 (2.1)	1 (1.6)	
Self-assessed economic status				
Lower	5 (3.7)	0 (0.0)	5 (2.8)	LR = 4.607 *p* = 0.203 *df* = 3
Lower middle	56 (41.5)	25 (53.2)	81 (44.5)	
Upper middle	69 (51.1)	21 (44.7)	90 (49.4)	
Upper	5 (3.7)	1 (2.1)	6 (3.3)	
Teaching hospital				
EKSUTH Ado-Ekiti	49 (36.3)	15 (31.9)	64 (35.2)	*χ*^2^ = 0.294 *p* = 0.588 *df* = 1
FETH Ido-Ekiti	86 (63.7)	32 (68.1)	118 (64.8)	
Type of training				
Internship	58 (43.0)	15 (31.9)	73 (40.1)	*χ*^2^ = 1.771 *p* = 0.183 *df* = 1
Residency	77 (57.0)	32 (68.1)	109 (59.9)	
Residency training (*n* = 109)				
Junior	56 (72.7)	11 (34.4)	67 (61.5)	*χ*^2^ = 14.039 *p* < 0.001* *df* = 1
Senior	21 (27.3)	21 (65.6)	42 (38.5)	
Level of job satisfaction				
Unsatisfied	29 (21.5)	4 (8.5)	33 (18.1)	*χ*^2^ = 11.522 *p* = 0.003* *df* = 2
Undecided	44 (32.6)	8 (17.0)	52 (28.6)	
Satisfied	62 (45.9)	35 (74.5)	97 (53.3)	

*LR* Likelihood ratio

^*^Statistically significant at *p* value < 0.05

The binary logistic regression of socio-demographic and other related factors associated with migration intention among doctors undergoing training in public tertiary hospitals in Ekiti State in is presented in Table 5. The determinants of migration intention include stage of the residency training (Senior OR: 0.24; 95% CI 0.09–0.67) and level of job satisfaction (Satisfied OR: 0.36; 95% CI 0.14–0.93).

**Table 5:** Determinants of migration intention among doctors undergoing training in Public Tertiary Hospitals in Ekiti State

Variables	Odd ratio	95.0% C.I	*p* value
Age group			
≤ 34 years (Ref)	1.0		
35 years and above	0.67	0.20–2.31	0.530
Marital status			
Single (Ref)	1.0		
Married	0.49	0.11–2.14	0.342
Residency training stage			
Junior (Ref)	1.0		
Senior	0.24	0.09–0.67	0.006*
Job satisfaction			
Unsatisfied/undecided (Ref)	1.0		
Satisfied	0.36	0.14–0.93	0.034*

* Statistically significant at *p* value < 0.05

## Discussion

The prevalence of migration intention, three in four among doctors undergoing residency and internship training programmes in the two public tertiary hospitals in Ekiti State is high. This is consistent with report by Hagopian et al. in Nigeria and Ghana where the majority of the students and doctors undergoing training across all the medical schools and tertiary hospitals in both countries had an intention to migrate abroad, at least to obtain more specialist training [21]. Also, findings from the present study are consistent with reports from similar studies among doctors undergoing training programmes in Pakistan [25], and among the final year students in Ethiopia [26] where the intention to migrate abroad for training was high. In a similar vein, findings in the present study is consistent with report by Syed et al. where the majority (95%) of final year medical students of Aga Khan University (AKU) and about two-thirds of final year medical students of Baqai University (BU) both in Pakistan intend to migrate abroad for their postgraduate training [27]. However, this is in contrast to what Goštautaitėa et al. [28] reported in Lithuanian, an European high-income country, where only one-fifth of doctors undergoing residency training, Stummer et al. [29] in Austria where less than half of the young doctors, and Ramos and Alves [24] in Portugal where about half of the junior doctors (doctors undergoing internship training) had intention to migrate to other developed nations. The implication of high intention to migrate abroad among doctors undergoing training in the public tertiary hospitals in Ekiti State may have potential disruptive effect on delivery of qualitative health care services to the people of the state and neighbouring states. Doctors undergoing internship and specialist training programmes form the critical physician workforce in the tertiary hospitals who render medical and surgical health services to clients and patients who daily seek care at the hospitals. Also, these findings imply that mobility of doctors especially the young doctors (resident and intern doctors) is a global phenomenon, although, it is worse in the lower- and middle-income countries compared with the high-income countries.

The present study reported very high initiation of plans (70%) towards actualization of their intent to migrate abroad among the doctors undergoing training programmes in the public tertiary hospitals in Ekiti State. This finding is consistent with what Humphries et al. in 2018 reported in a qualitative survey among Irish early career doctors where 60% of those intending to migrate abroad have immediate plans to do so [30]. The qualitative nature of the Irish survey implies methodological differences from the present study among the respondents in public tertiary hospitals in Ekiti State. In a similar vein, study among medical students in Serbia by Santric-Milicevic et al. reported that majority (about three-fifths) of the respondents were either at an advanced planning phase or had a firm plan in place to migrate abroad following completion of their medical education programme [31].

The findings from the present study show that there is a statistically significant relationship, both at the bivariate and multivariate analysis levels, between job satisfaction among doctors undergoing residency and internship training in the two public tertiary hospitals in Ekiti state and their intention to migrate abroad to practice. Majority of the doctors undergoing training who were satisfied with their job do not intend to migrate abroad, compared with those who were unsatisfied and undecided on their level of job satisfaction. This is consistent with findings by Opoku and Apenteng 2014 in Ghana where the doctors undergoing internship training or medical officers and doctors who reported dissatisfaction with their compensation were more likely to report that they had intention of migrating away from Ghana within the next 5 years [32]. Also, studies in Poland [33] and Ireland [34] reported that overall level of career satisfaction was negatively related to the doctors’ intention to migrate abroad. There are few studies that have explored the relationship between migration intention among medical doctors and much less among doctors undergoing training. Similar to our findings which established a strong relationship between intention to migrate abroad and job satisfaction, Jadoo et al. in a study among Iraqis doctors also reported that intention to quit present employment was associated with low job satisfaction score [12]. The implication of finding of inverse relationship between job satisfaction among undergoing training and their intention to migrate abroad include the fact that hospital management and policy-makers can address the high intention to migrate abroad among this group of doctors by improving the various dimensions of job satisfaction such as work environment, remuneration, recognition on the job, training and skill acquisition.

The stage of residency training programme (RTP) being a determinant of intention to migrate abroad among the trainee doctors is a major finding in our study, barely reported in previous published studies. The doctors in the senior RTP have lower probability of intention to migrate abroad compared with those in the junior RTP. This finding may be due to a higher level of commitment to the Nigerian health system by the doctors in the senior RTP, having successfully navigated the more rigorous and wider in scope junior RTP.

This study has few limitations. This study is a cross-sectional study as such, temporality may not be ascertained. The self-reported nature of the responses might introduce bias such as social desirability bias. This was minimized by assuring the respondents of absolute confidentiality of information provided and the questionnaire did not contain individual personal identities. Also, findings from this study cannot be generalized to all doctors working in the Nigerian health system, as only a section of them are undergoing training programmes.

## Conclusion

The prevalence of intention to migrate abroad among the doctors undergoing training in public tertiary hospitals in Ekiti State is high, with the majority already working on plans to migrate abroad. The major determinants of migration intention are the stage of residency training and level of job satisfaction. If this high level of intention to migrate translates to actual migration, it will affect the retention of this critical health workforce in the public tertiary hospital settings in the state and may portend serious implication on the standard and quality of health care delivery to the people who seek health care services in the two major health institutions in the state.

## Recommendations

The authors therefore recommended that there is a need to consider the implications of doctors’ migration abroad from the tertiary hospitals on the quality of healthcare services available to the people in the state and its environs. As such, individual doctor intending to migrate abroad should consider his/her decision on its merit, not just as a fad that should be followed. Also, the management of each hospital should develop retention strategies for human resources for health in their establishment to avert the possibility of losing their trainee doctors, whom a lot have been invested in, to other nations. Some of the retention strategies that have worked among trainee doctors include improved remuneration and welfare packages, good working climate with up-to-date medical equipment, adequate funding of residency training programme, incorporating further specialist training opportunities both locally and abroad, industrial harmony among peers, seniors and other cadres in the hospital, among others. There is a need for consultant trainers and hospital management to provide more and better support to the doctors in junior RTP to engender seamless transition to the senior RTP.

## Data Availability

Datasets supporting the findings of this study are available from the corresponding author, A.A.F., on request.
